# Durum wheat heat tolerance loci defined via a north–south gradient

**DOI:** 10.1002/tpg2.20414

**Published:** 2023-12-07

**Authors:** Amadou Tidiane Sall, Hafssa Kabbaj, Sidi ould Ely Menoum, Madiama Cisse, Mulatu Geleta, Rodomiro Ortiz, Filippo M. Bassi

**Affiliations:** ^1^ Centre de Recherches Agricoles Institut Sénégalais de Recherches Agricoles (ISRA) Saint‐Louis Senegal; ^2^ International Center for the Agricultural Research in the Dry Areas (ICARDA) Rabat Morocco; ^3^ Centre National de Recherche Agronomique et de Développement Agricole (CNRADA) Kaédi Mauritania; ^4^ Department of Plant Breeding Swedish University of Agricultural Sciences (SLU) Lomma Sweden

## Abstract

The global production of durum wheat (*Triticum durum* Desf.) is hindered by a constant rise in the frequency of severe heat stress events. To identify heat‐tolerant germplasm, three different germplasm panels (“discovery,” “investigation,” and “validation”) were studied under a range of heat‐stressed conditions. Grain yield (GY) and its components were recorded at each site and a heat stress susceptibility index was calculated, confirming that each 1°C temperature rise corresponds to a GY reduction in durum wheat of 4.6%–6.3%. A total of 2552 polymorphic single nucleotide polymorphisms (SNPs) defined the diversity of the first panel, while 5642 SNPs were polymorphic in the “investigation panel.” The use of genome‐wide association studies revealed that 36 quantitative trait loci were associated with the target traits in the discovery panel, of which five were confirmed in a “subset” tested imposing heat stress by plastic tunnels, and in the investigation panel. A study of allelic combinations confirmed that Q.icd.Heat.003‐1A, Q.icd.Heat.007‐1B, and Q.icd.Heat.016‐3B are additive in nature and the positive alleles at all three loci resulted in a 16% higher GY under heat stress. The underlying SNPs were converted into kompetitive allele specific PCR markers and tested on the validation panel, confirming that each explained up to 9% of the phenotypic variation for GY under heat stress. These markers can now be used for breeding to improve resilience to climate change and increase productivity in heat‐stressed areas.

## Core Ideas

- The use of a north–south gradient between the Senegal River and Morocco was introduced to assess heat tolerance.
- Critical loci, involved in the control of heat tolerance in durum wheat, was defined.
- Germplasm sources and loci tagged by kompetitive allele specific PCR to favor the introgression of heat tolerance alleles by breeding were presented.
- A new gold standard by using three germplasm panels to discover, investigate, and validate useful loci was studied.

**Abbreviations**
ANOVA: analysis of variance
Biom: biomass
BLUE: best linear unbiased estimator
DtH: days to heading
DtM: days to maturity
FAN: Fanaye
GDP: Global Durum Panel
GFP: grain filling period
Gr·spk: grains per spike
GWAS: genome‐wide association study
GY: grain yield
HSI: heat susceptibility index
ICARDA: International Center for Agricultural Research in the Dry Areas
KASP: kompetitive allele specific PCR
KED: Kaedi
LD: linkage disequilibrium
LOD: logarithm of the odds
MAS: marker‐assisted selection
MKZ: Melk Zhar
MTA: marker‐trait association
PCA: principal component analysis
QTL: quantitative trait locus
SNP: single nucleotide polymorphism
TES: Tessaout
TKW: 1000‐Kernel weight

## 1 INTRODUCTION

Durum wheat (*Triticum durum* Desf.) is grown on over 17 million ha worldwide and accounts for around 6% of total wheat production (Sall et al., 2019). Durum wheat is a staple crop in North Africa and West Asia (Elias & Manthey, 2005) and it is slowly becoming a strategic crop in sub‐Saharan Africa and South Asia (Sall et al., 2019). Heat is one of the most important abiotic stresses affecting this crop, with an estimated 58% of global production undergoing mild to severe losses due to high temperatures each year (Kosina et al., 2007). For instance, in France, the wheat yield has been predicted to suffer a 3.5%–12.9% reduction in the medium term due to the rise in temperatures (Wang et al., 2018). In China, researchers reported that wheat productivity could be reduced by 3%–10% with just 1°C increase in temperature during the season (You et al., 2009). Similar predictions are also supported by Eruygur and Özokcu (2016), suggesting the loss of 8%–23% of global wheat production by the end of 2100. Furthermore, temperatures are expected to rise soon due to climate change (Intergovernmental Panel on Climate Change [IPCC], 2018; Ortiz et al., 2008). Hence, technological solutions must be converted rapidly into actions. Breeding heat‐tolerant crops remains one of the most viable options to maintain adequate production. For that, a key step is the identification of the loci responsible for the adaptive response of tolerant genotypes. Once identified, these loci can then be pyramided using targeted crossing and marker‐assisted selection (MAS) schemes. Genome‐wide association study (GWAS) is a valuable tool to pinpoint the genomic regions involved in the control of useful traits, even for traits with complex inheritance such as grain yield (GY) under stress (Charmet et al., 2009; Crossa et al., 2007; Maccaferri et al., 2011; McIntyre et al., 2010; Rehman‐Arif et al., 2012). In durum wheat, quantitative trait loci (QTLs) for heat tolerance have been identified on chromosomes 1B, 2B, 3A, 3B, 5A, 5B, 7A, and 7B (Awlachew et al., 2016; El Hassouni et al., 2019; Maccaferri et al., 2011; Mohammadi et al., 2008). However, their deployment in breeding has been thus far limited (F. M. Bassi et al., 2023). Further, the environments utilized to investigate the heat stress did not represent the expected dreadful scenario that climate change will impose by the end of the century.

**Core Ideas**

- The use of a north–south gradient between the Senegal River and Morocco was introduced to assess heat tolerance.
- Critical loci, involved in the control of heat tolerance in durum wheat, was defined.
- Germplasm sources and loci tagged by kompetitive allele specific PCR to favor the introgression of heat tolerance alleles by breeding were presented.
- A new gold standard by using three germplasm panels to discover, investigate, and validate useful loci was studied.

Recently, a panel of durum cultivars and elite breeding lines (Kabbaj et al., 2017) was evaluated for 2 years at two sites under extremely warm conditions along the Senegal Basin, where the maximum daily temperature remains above 30°C throughout the growing season (Sall, Kabbaj, et al., 2018; Sall, Bassi, et al., 2018). Several entries have been identified as showing good response to this severe heat stress. By defining the genomic region associated with the heat tolerance identified in the durum wheat genotypes, the work presented here aimed to further advance the results obtained in that study. To that scope, a north–south heat gradient was implemented by combining the field performances along the Senegal River (Sall, Bassi, et al., 2018) with those obtained from field trials in Morocco, 14 latitude parallels further North. A small subset of the same panel was also tested by applying heat stress at the time of flowering and imposing plastic tunnels to raise temperatures by up to 10°C (El Hassouni et al., 2019). The most critical QTLs were then first discovered by performing GWAS in the larger “discovery panel,” then further confirmed by allelic combination study in a different “investigation panel,” and ultimately the closely linked single nucleotide polymorphisms (SNPs) were converted into kompetitive allele specific PCR (KASP) technology and “validated” on a third panel. These KASPs are now ready to be deployed by MAS across durum wheat breeding programs to improve its heat tolerance globally.

## 2 MATERIALS AND METHODS

### 2.1 Plant material

The present study was conducted using three germplasm panels. The first panel was defined as “discovery panel” and it includes 216 modern durum wheat lines (Table S1) from different countries selected from a larger panel of 384 entries described by Kabbaj et al. (2017). Because of the unique conditions of the test environments, only 216 entries reached field maturity and could therefore be used. Further, a “subset” of this panel comprising 42 modern durum wheat lines selected for their similarity in flowering time and genetic diversity was used for a plastic tunnel screening as described by El Hassouni et al. (2019). The full panel was further used to assess all loci associated to the control of phenology across 13 environments (Gupta et al., 2020). A second germplasm set defined as “investigation panel” included 144 advanced durum wheat elite lines (Table S2) from the breeding program of the International Center for Agricultural Research in the Dry Areas (ICARDA). These elite lines were selected because at least one of the top heat tolerant entries identified by Sall, Bassi et al. (2018) via phenotypic screening of the discovery panel was incorporated into their pedigree. The third set of entries defined as “validation panel” included 85 ICARDA's elite lines (Table S3) that constituted the 40th International Durum Observation Nurseries, which were provided to over 50 partners around the world for testing.

### 2.2 Field stations

Two irrigated field stations were used for phenotyping in the South of Morocco: Melk Zhar (MKZ; 30°02″ N, 9°48″ W) during seasons 2014–2015 and 2015–2016 and Tessaout (TES; 29°83″ N, 8°57″ W) during season 2015–2016. MKZ has sandy soil with low water holding capacity, while TES has red silt soil with high water holding capacity. In addition, the germplasm was grown under the hot‐irrigated savanna conditions of the Senegal River basin at two stations (Kaedi [KED] and Fanaye [FAN]) and details on the growing conditions along the Senegal River can be found in Sall, Kabbaj, et al., 2018; Sall, Bassi, et al., 2018). Briefly, KED in Mauritania (KED, 15°99″ N, 13°07″ W) and FAN in Senegal (FAN, 16°53″ N, 15°22″ W) were used during 2014–2015, 2015–2016, 2018–2019, and 2019–2020 seasons. A total of 14 latitude parallels separates the 30° N of the Moroccan stations from the 16° N parallel of the Senegal River stations, creating a substantial north–south gradient that mostly generates a difference in temperature caused by the different proximity to the tropics. Instead, only four longitudinal parallels separate the 9° W of the Moroccan stations from the 13° W of the Senegal River stations, reducing the effect of variation in sunlight exposition (i.e., same time zones). For simplicity, each season is indicated with its second year (i.e., 2015–2016 is expressed as “16”). FAN has sandy‐clay soil with higher organic matter and good water holding capacity, while KED has lighter sandy‐loam‐clay soils with intermediate water holding capacity. At all locations, irrigation was guaranteed to avoid any moisture stress. In MKZ during the 2014–2015 season, 26 irrigations (twice per week for 13 weeks) of 13 mm each were applied using dripper pipes in addition to the 297 mm of rainfall for a total moisture of 635 mm. During season 2015–2016, the number of irrigations was increased to 30 to compensate for the extremely low rainfall (47 mm) to achieve a total of 437 mm of moisture. In TES, nine gravity irrigations were provided for an estimated amount of 360 mm of water in addition to 95 mm of rainfall for a total of 455 mm. In KED, gravity irrigations were performed every 7 days for 10 irrigations in total over a 3‐month season, which provides a total moisture amount of 320 mm. In FAN, the same number of irrigations was applied except that the estimated total moisture provided by each was slightly higher for a total of 380 mm. At all stations, the same amount of fertilizer was provided: pre‐sowing 50 units of nitrogen (N), phosphate, and potassium, followed by two splits of 50 units of N each before and after flowering. Only in MKZ, the additional 100 units of N were applied in four splits of 25 units each. Planting was conducted during the third and fourth week of November at MKZ and TES, while it was carried out during the second week of December along the Senegal River.

### 2.3 Phenotypic characterization

The discovery panel was tested in seven environments: MKZ two seasons (15 and 16), TES one season (16), and KED and FAN two seasons (15 and 16). The subset of the discovery panel was tested as explained in El Hassouni et al. (2019), applying plastic tunnels at the time of flowering to increase the temperatures by up to 15°C. The investigation panel was tested at FAN during the 19 and 20 crop seasons. The validation panel was tested at FAN during the 19 crop season. All panels were field‐tested in a partially replicated (augmented) design with blocks of size 24, each including four replicated checks. The checks were Omrabi 5, Icarasha 2, Azeghar2, and Waha. The plot planting surface was of 3‐m length × 5 rows spaced 30 cm apart (4.5 m^2^) at a sowing density of 120 kg ha^−1^.

Phenology traits, yield components, and GY were recorded for all genotypes. The days to heading (DtH) was recorded as the number of days elapsed from sowing to the moment that 50% of the plot showed spikes emerging from the flag leaf at stage 59 in the Zadoks scale (Z59, Zadoks et al., 1974). Before maturity (Z83‐87), the number of fertile spikes per meter square (Spk·m^2^) was counted. Days to maturity (DtM) was recorded when 50% of the spikes turned yellow (Z91‐92). The length of the grain filling period (GFP) was then computed as the difference between DtM and DtH. Plant height was measured in centimeters from the ground to the top of a representative spike excluding its awns. For each plot, only the middle rows that cover an area of 2.7 m^2^ were harvested, dried, and the biomass (Biom) weighted before threshing. The weight of the threshed grains was converted into GY expressed as kg ha^−1^. The ratio between the GY and Biom was expressed as harvest index. One thousand grains were weighted in grams as 1000‐kernel weight (TKW). The number of grains per square meter (Gr·m^2^) was calculated using the weight of the grains harvested from 2.7 m^2^ area and the weight of one kernel derived from the TKW value as follows:

$$\;\mathrm{Gr}\cdot m^{2}=\;\frac{\mathrm{Harvested}\;\mathrm{weight}\;\mathrm{of}\;\mathrm{plot}}{2.7\;m^{2}\;\times \;\frac{\mathrm{TKW}}{1000}}$$

The number of grains per spike (Gr·spk) was derived from dividing the calculated number of grains per unit area by the number of spikes recorded for the same area as follows:

$$\mathrm{Gr}\cdot \mathrm{spk}\;=\;\frac{\;\mathrm{Gr}\cdot m^{2}}{\mathrm{Spk}\cdot m^{2}}$$

### 2.4 Statistical analysis of phenotypic data

Each location for each season was defined as a single environment, thereby resulting in nine environments (KED15, KED16, FAN15, FAN16, FAN19, FAN20, MKZ15, MKZ16, and TES16). Analyses of variance (ANOVAs) were performed, and best linear unbiased estimators (BLUEs) were obtained for each environment individually using the "lme4" package (Bates et al., 2015) in R Studio V 4.0.1 (R Development Core Team, 2020). The climatic information for maximum and minimum temperatures was collected from weather stations located at the research farms. The average maximum and minimum temperatures were then calculated for each major plant stage (vegetative stage, tillering, flowering time, and GFP) for each environment. This climate matrix was then used to conduct regression analysis against GY to determine the most significant climatic factor affecting yield performance of the germplasm. Only using those climatic factors with a significant influence on GY, the environments were clustered using "Dcluster" package (Gomez‐Rubio et al., 2005) in R Studio V 4.0.1, via hierarchical clustering based on Euclidean distance determined through principal component analysis (PCA). Within these clusters of environments, there is still a chance to encounter unaccounted GxE effect. For that reason, combined ANOVAs were performed for the discovery panel using BLUE, considering all sources of variations as fixed effects across all environments and each climatic clusters of environments determined by PCA. The BLUES for each climatic cluster were then used to determine genotypes tolerant to heat stress by implementing the heat susceptibility index (HSI) as suggested by El Hassouni et al. (2019) by the following equation:

$$\mathrm{HSI}\;=\frac{1-\frac{Ys}{Yp}}{1-\frac{.Ys}{.Yp}}$$

where *Y*s and *Y*p are the values obtained under temperature stressed (s) and non‐stressed (p) conditions, respectively, and .*Y*s and .*Y*p are the mean values of all lines for the specific trait obtained under stress and non‐stress conditions, respectively. Based on environmental clustering, the Moroccan sites were considered as non‐stressed (p), while those of Senegal River as heat stressed (s). HSI was determined for GY, TKW, and Gr·spk. The individuals achieving an HSI score of less than 0.70 were considered as heat “tolerant,” while values of less than 1.2 as “non‐tolerant,” and scores above 1.2 defined “susceptible” entries.

### 2.5 Genotyping, population structure, and linkage disequilibrium pattern analysis

For the discovery panel, a complete description of the genotyping methodology and definition of population kinship was previously presented in Kabbaj et al. (2017). Briefly, the Axiom 35K array was used to screen entries of the discovery panel. The sequences of these markers were aligned to the Svevo genome assembly (Maccaferri et al., 2019) using a cutoff of above 97% sequence identity. A total of 7652 high‐quality polymorphic SNPs with minor allele frequency of above 5% were retained for downstream analysis (Table S1). However, for the specific study presented here, only 216 entries reached field maturity, since most of the landraces failed to flower or reach ripening along the Senegal River. Within this subset, only 2552 SNPs met all cutoff conditions. A selection of 500 highly informative (polymorphic information content > 0.25) and evenly spaced markers were used to determine that modern lines of this panel are separated into six sub‐groups representing the breeding programs of origin. Linkage disequilibrium (LD) decay was estimated using the “Neanderthal” method (Jujumaan, 2017) at 51.3 Mbp as presented in F. Bassi et al. (2019) for the discovery panel. The investigation panel was genotyped with a 23K array chip developed by SGS–Institut Fresenius Trait Genetics section, which incorporates 14.5K SNPs from the 90K Infinium Array, 8.5K SNPs from the Axiom array, and 265 SNPs that were reported to be linked to genes (Table S2). Marker curation was conducted for the discovery panel, which resulted in 5642 polymorphic SNPs. These were also aligned to the Svevo genome assembly (Maccaferri et al., 2019), and linkage analysis revealed that the LD decay was 36.3 Mbp. Kinship analysis by STRUCTURE identified three clusters for the investigation panel.

KASP assays were designed by LGC Genomics by providing the sequences of the most representative array probes underlying the QTL of interest. The KASP assays comprised three primers (one common reverse primer and two primers for each of the two alleles) for each array probe. These were then used to screen the validation panel. All genotyping was done using LGC property complete genotyping service (Table S3). The primer sequences of the markers are protected by commercial rights and cannot be disclosed here. However, they can be purchased by users as a service from LGC by providing the marker names given here as references.

### 2.6 Genome‐wide association study

GWAS was carried out using TASSEL 5.0 software (Bradbury et al., 2007), using the mixed linear model, population structure coefficients (*Q*), and kinship matrix (K) for the discovery panel. While the generalized linear model was used for investigation panel as it better fits the data. DTH was used as a covariate for all studies to protect against the rediscovery of major flowering loci. The traits GY, TKW, and Gr·spk were investigated via GWAS at each environment individually and, as combined across environments, also using HSI. The significance of marker‐trait association (MTA) was assessed by Bonferroni revised equation as suggested by Duggal et al. (2008), using a very stringent threshold of 0.01. Hence, the significant logarithm of the odds (LOD) threshold was set at 2.69 and 2.83 for the discovery panel and investigation panel, respectively. MTAs located within less than two times the LD decay distance were considered as underlying the same haplotype block and hence merged into one QTL. For correction, regression analysis was performed between haplotype scores of flanking markers under each QTL. Flanking markers with values *r* ^2^ < 0.2 were considered unlinked and therefore new QTL boundaries were defined. Pearson's critical value (Pearson, 1985) for correlation was squared to obtain a critical *r* ^2^ = 0.032 at *p* < 0.05 for both panels, which was used as the threshold to determine QTL that explained a significant part of the phenotypic variance. To further remove the effect of phenology from QTL discovery, any QTL overlapping with a significant MTA identified for DTH was removed from all downstream considerations (Gupta et al., 2020). Allelic combination analysis was conducted on the discovery panel to define which QTLs were additive in nature. For each QTL, the marker with the highest LOD against GY was selected to represent the allelic status of each genotype. Classes of alleles at the main QTL were defined for each genotype. A two‐way ANOVA and the least significant difference test were used to determine significantly superior classes of alleles using genotypes within the same class of allele as replications. Those QTLs that were identified in the same genomic region by the discovery panel, and its subset using plastic tunnels (El Hassouni et al., 2019), as well as in the investigation panel were considered the most critical and were then further validated using KASP method.

### 2.7 Markers validation by KASP

The conversion of Axiom markers to KASP markers was achieved by submitting the sequences of the probes on the array associated with the desirable traits to LGC Genomics for in silico designing of KASP primers using their proprietary software. Those designs that passed the in silico criteria were purchased and used to genotype the validation panel. For each amplified marker that showed polymorphism, the correlation significance cutoff between the phenotype and allelic score was set at *r* = 0.105 following Pearson's critical value for *p* < 0.05 (Pearson, 1985). In addition, the top 20 and lowest 20 lines in terms of GY in FAN19 were considered as the true positive and true negative, respectively. Hence, the accuracy was calculated as the ratio of the correct allelic call among all, sensitivity as the ratio of the correct positive allelic calls among the top 20 high‐yielding lines, and specificity as the ratio of the correct negative allelic calls among the 20 lowest yielding lines.

## 3 RESULTS

### 3.1 A north–south heat gradient

There are 14 latitude parallels and only four longitudinal parallels that separate the Moroccan stations (MKZ and TES) from the two stations along the Senegal River of KED (Mauritania) and FAN (Senegal). This results in an average temperature difference of 10°C during the crop season, but only a limited change in the daily sun exposure (Table 1). The average minimum and maximum temperatures across the season at the stations located along the Senegal River basin were 17°C and 35°C, respectively, while the average minimum and maximum temperatures at the Moroccan stations were 8°C and 22°C, respectively. Temperatures varied across the sites and years with warmer temperatures during the season 16, mainly during the flowering windows. The maximum temperature at flowering in TES station was 21°C during the season 16; in MKZ, it was 22°C; in FAN, it was 37°C; and in KED, it was 39°C. Furthermore, at all the sites, the moisture was kept beyond the level of stress for the crop to complete the cycle, making temperature the main limiting factor for the plants.

**TABLE 1:** Average minimum and maximum temperature during different stages of the crop growth (vegetative, tillering, flowering, and grain filling) across the tested environments, ordered by hierarchical clustering for temperature.

			Average minimum temperature (°C)				Average maximum temperature (°C)			
Station	Season	Cluster^a^	Vegetative	Tillering	Flowering	Grain filling	Vegetative^*^	Tillering^*^	Flowering^*^	Grain filling^*^
Tessaout	2015–2016	Non‐stressed	5	5	5	11	23	20	21	27
Melk Zhar	2014–2015	Non‐stressed	8	6	7	12	21	21	20	24
Melk Zhar	2015–2016	Non‐stressed	8	8	8	12	24	21	22	23
Fanaye	2018–2019	Heat‐stressed	12	12	16	15	32	32	35	33
Fanaye	2015–2016	Heat‐stressed	14	15	14	18	32	33	37	36
Kaedi	2014–2015	Heat‐stressed	22	23	18	24	33	35	41	38
Kaedi	2015–2016	Heat‐stressed	21	22	22	23	32	34	39	37
Fanaye	2019–2020	Heat‐stressed	14	14	14	15	32	31	37	37
Fanaye	2014–2015	Heat‐stressed	15	16	15	15	31	32	38	33

^a^ Clustering by principal component analysis (PCA) using significant climatic factors influencing grain yield.

* *p* < 0.01, highly significant effect on grain yield.

The average maximum and minimum temperatures were recorded for each crop stage (vegetative, tillering, flowering, and grain filling) for each environment, and these were regressed against GY (Table S4). The maximum temperature was the most critical factor affecting GY at all stages of the crop. The clustering pattern of the nine environments (Figure S1) was then determined through PCA based on the significant climatic factors: maximum temperature stress during the vegetative, flowering, and grain filing stages. MKZ 15 and 16 were clustered together with TES 16 constituting the non‐heat stressed environments, whereas the other six environments along the Senegal River formed the second cluster, which was defined as the heat‐stressed cluster.

### 3.2 Yield component performance under north–south heat gradient

The effect of temperature led to highly significant differences (*p* < 0.001) for genotype and genotype × environment effects for GY, TKW, and Gr·spk across sites (Table S5). As expected, the site exposed to the highest temperatures (FAN16) yielded the least with an average GY of 1927 kg ha^−1^, whereas the highest GY of 8710 and 8890 kg ha^−1^ were recorded at TES and MKZ, respectively (Figure S2). The top GY achieved in the heat stressed environments were 4800 and 5700 kg ha^−1^ at KED 15 and FAN 15, respectively, despite a condensed crop season of just 95 days. The average GY at FAN was 35% and 52% lower compared to MKZ in 15 and 16, respectively, and 63% less compared to TES 16. In KED, the average yield was reduced by 35% and 46% compared to MKZ and by 59% compared to TES 16. The regression at each environment against GY confirmed that Grn·spk was the most critical trait to ensure higher GY under heat‐stressed conditions (Table 2).

**TABLE 2:** Regression (*r* ^2^) for days to heading (DtH), grains per spike (Gr·Spk), 1000‐kernel weight (TKW) against grain yield (GY) at each environment and average across cluster 1 of non‐stressed environments and cluster 2 of heat‐stressed environments.

	Environment	Regression GY (*r* ^2^)	
Environment	DtH	Gr·Spk	TKW
TES16	0.00	0.00	0.01
MKZ15	0.00	–	0.01
MKZ16	0.10^**^	0.00	0.29^*^
Combined 1	0.03^**^	0.00	0.10
FAN15	0.10^**^	–	0.04^*^
FAN16	0.14^**^	0.04^*^	–
FAN19	0.01	0.35^**^	–
FAN20	–	0.41^**^	−0.01
KED15	0.08^**^	–	0.05^*^
KED16	0.01	0.53^**^	–
Combined 2	0.06^**^	0.33^**^	0.02

Abbreviations: 15, season 2014–2015; 16, season 2015–2016; 19, season 2018–2019; 20, season 2019–2020; FAN, Fanaye; KED, Kaedi; MKZ, Melk Zhar; TES, Tessaout.

^*^ *p* < 0.05; ^**^ *p* < 0.001.

### 3.3 Heat susceptibility index

**FIGURE 1:**
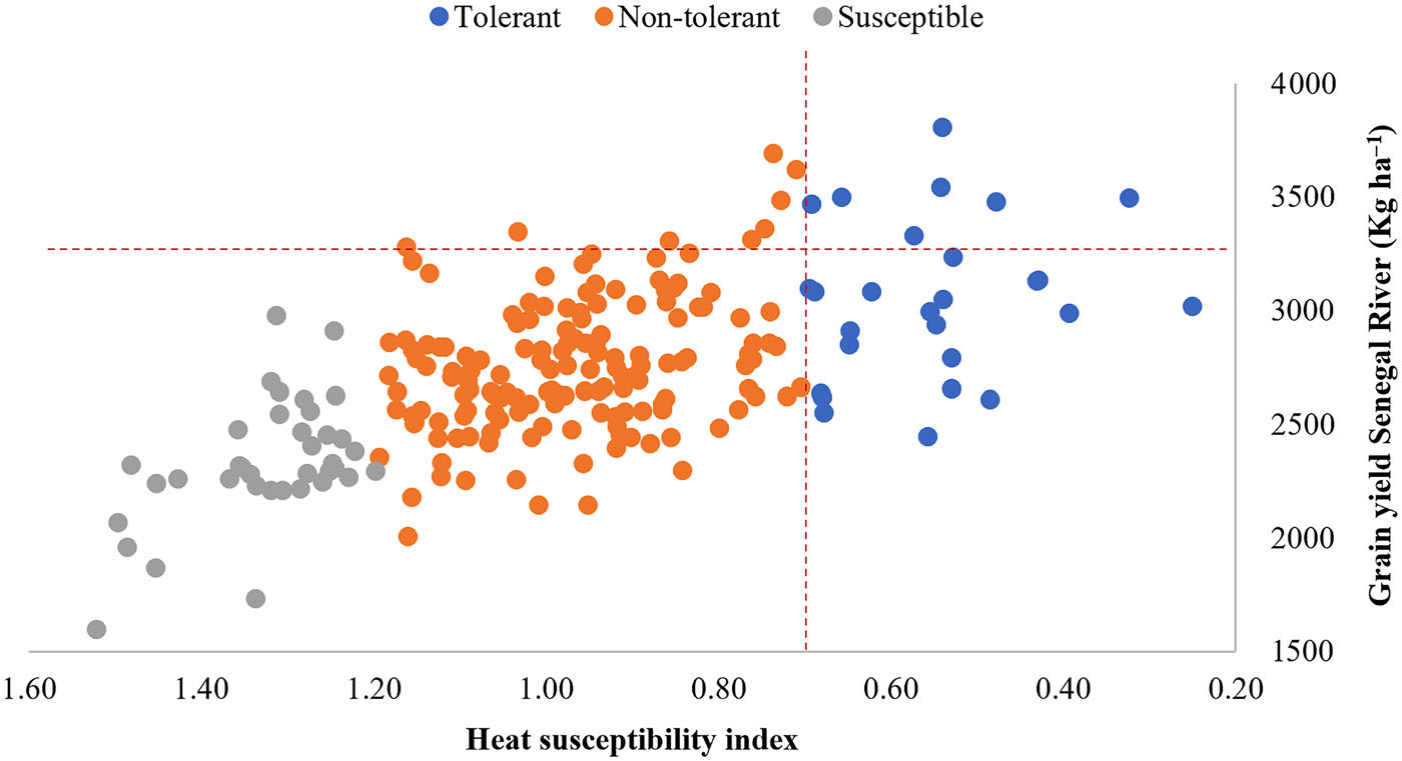
Heat tolerance response of entries tested across locations for heat susceptibility index (HSI) versus grain yield combined across heat stressed environments of the Senegal River. Color coding is based on the HSI. The dashed lines mark the boundaries for "tolerant" HSI and top 10% grain yield.

An HSI was defined based on the contrasting performances of the entries across the two heat‐stressed (KED and FAN) and non‐stressed (TES and MKZ) clusters, knowing that the temperature difference between the clusters was up to 10°C before flowering and up to 12°C after flowering. Since good HSI scores can be also achieved by very low‐yielding entries, this score was plotted against the GY calculated across all heat‐stressed environments (Figure 1). Twenty‐seven lines (12.5% of total) were identified as heat tolerant, with the Australian cultivar Wollaroi achieving the overall best HSI = 0.25 (Table S1). Intersecting combining HSI scores with GY confirmed three ICARDA's elites as the best performers, namely, Berghouata1 (3806 kg ha^−1^ and HSI = 0.54), ADYT_097 (3545 kg ha^−1^ and HSI = 0.54), and Arislahn5 (3500 kg ha^−1^ and HSI = 0.66).

### 3.4 Marker‐trait associations of the discovery and investigation panel

A GWAS was carried out for GY, TKW, and Gr·spk at each environment individually to identify 1144 MTAs. GWAS was also performed for the HSI for the discovery panel. The significant MTAs identified were merged into 36 significant QTLs spread across all durum wheat chromosomes (Table S6), after removing any QTL associated with the control of phenology (Gupta et al., 2020). Comparing these QTLs to those identified in the plastic tunnel heat stress study run by El Hassouni et al. (2019) confirmed 17 QTLs as cross‐validated.

**FIGURE 2:**
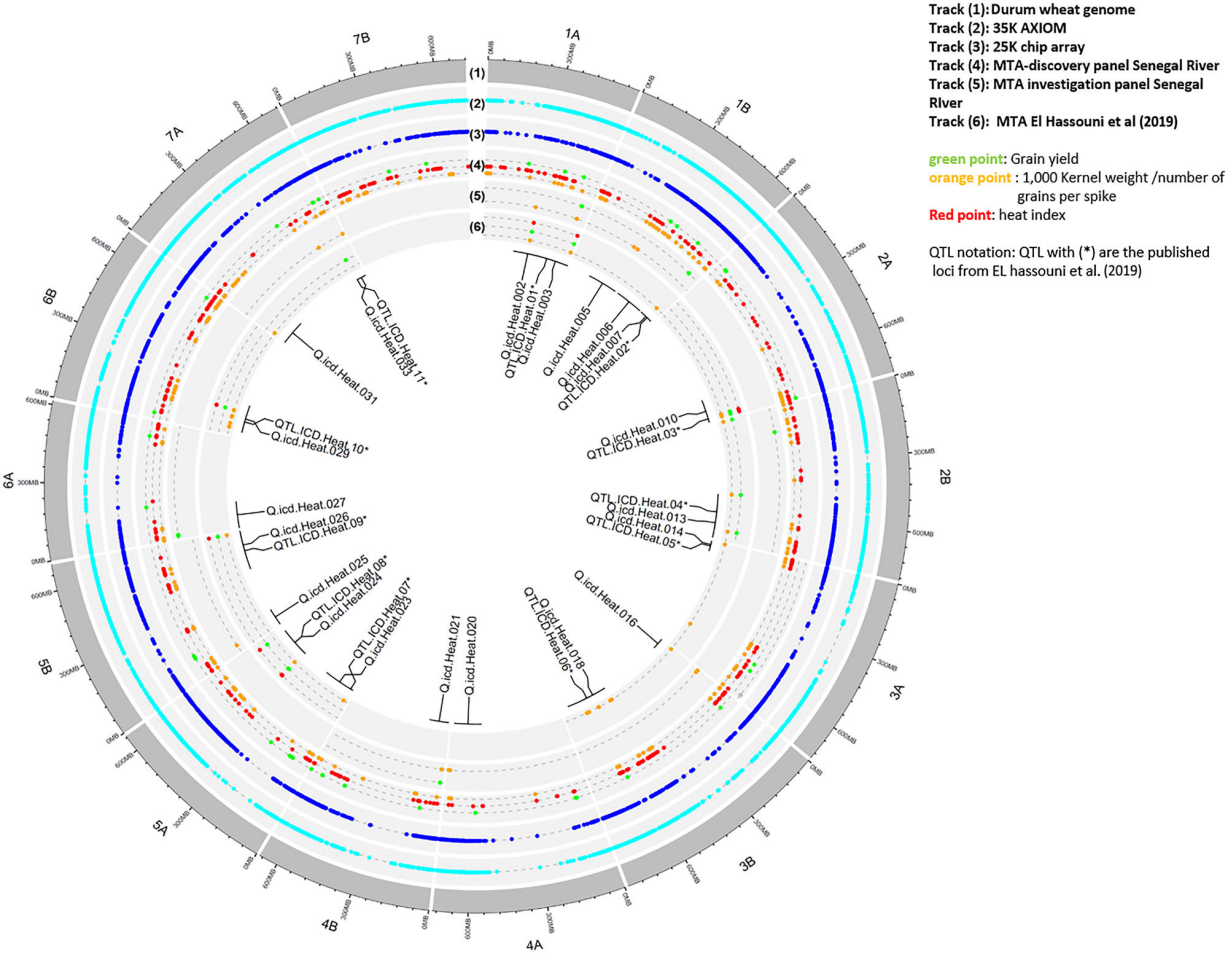
CIRCOS representation of the marker‐trait association aligned to the durum wheat genome based on the physical locations of the 35K Axiom array and 25K chip array probes, including the "discovery" and "investigation" panels, and those reported in El‐Hassouni et al. (2019). Quantitative trait locus (QTL) names are reported in the innermost circle with “*” placed next to those identified in all three studies.

Conducting GWAS on the investigation panel confirmed 11 of the initial 36 loci, of which five QTLs were also identified in the previous work by El Hassouni et al. (2019) (Figure 2). These five consistent QTLs were identified on the long arm of chromosomes 1A (Q.icd.Heat.003) and 7A (Q.icd.Heat.033), on the short arm of 2B (Q.icd.Heat.011) and 3B (Q.icd.Heat.016), and in the pericentromeric region of 1B (Q.icd.Heat.007). Interestingly, all these five loci were associated with GY, but also to TKW, Gr·spk^−1^ and their HSIs confirm strong interactions among these traits for heat tolerance (Table S6).

### 3.5 Multi‐QTL allelic combination additive effects

**FIGURE 3:**
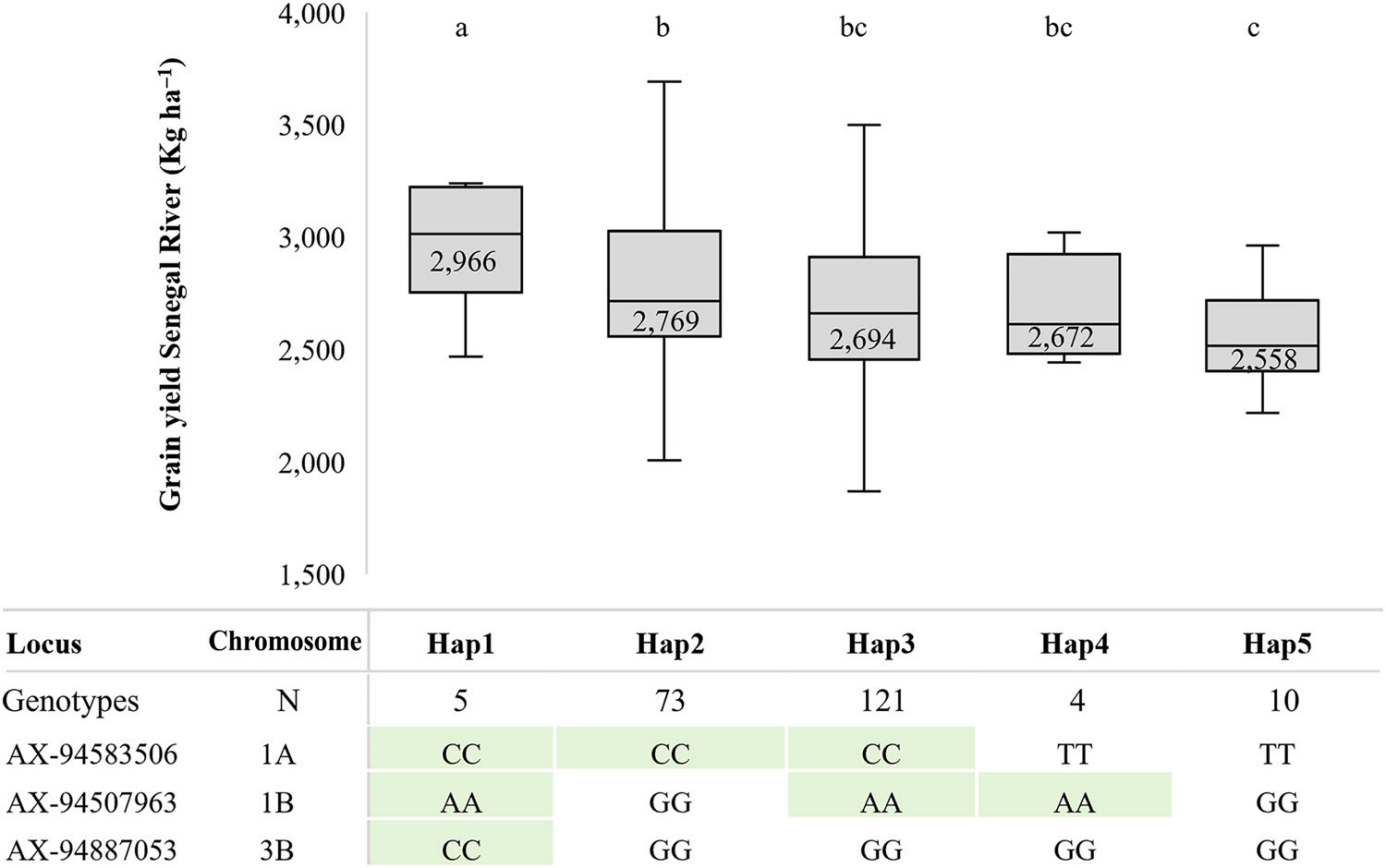
Allelic combination analysis for three major quantitative trait loci (QTLs) involved in grain yield based on the allele of their most representative marker: Q.icd.Heat.003 chromosome 1A, Q.icd.Heat.007 chromosome 1B, and Q.icd.Heat.016 on chromosome 3B. The box whiskers represent the quartiles of the performances for grain yield combined across the Senegal River heat stressed environments for entries of the "discovery panel" segregated by their allelic class. The actual average is reported inside the box as kg ha^−1^. The significant classes based on least significant difference (LSD) are reported above the boxes as letters. The positive allele is identified with a green background in the allelic table. The number of genotypes per class (*N*) is also reported.

Allelic combination analysis was carried out for the three QTLs (Q.icd.Heat.003, Q.icd.Heat.007, and Q.icd.Heat.016) influencing GY (Figure 3). Five multi‐loci allelic classes were identified that resulted in a significant (*p* < 0.05) effect on GY calculated across the heat‐stressed environments along the Senegal River. The combination of three positive alleles (Hap1: CAC) resulted in the highest average GY (2966 kg ha^−1^), while the absence of any positive allele (Hap5: TGG) resulted in the lowest average GY (2558 Kg ha^−1^) equal to a reduction of 16% (−408 kg ha^−1^). The most critical QTL appears to be Q.icd.Heat.016 on chromosome 3B, since not carrying the positive allele here caused a GY reduction of 197 kg ha^−1^ (8%) even when positive alleles at the other two QTLs remained. Q.icd.Heat.007 on chromosome 1B appears as the least critical, since lines that carried only the positive allele for it had average GY non‐significantly different from those not having any positive alleles. Interestingly, the top performing elites Berghouata1 and ADYT_097 have the Hap2 type, while Arislahn5 has the Hap3. Among the best eight entries, only the ICARDA's elite Icavicre harbors Hap1 and it achieved good performances with GY of 3237 kg ha^−1^ and an HSI of 0.53.

### 3.6 Markers validation by KASP

**FIGURE 4:**
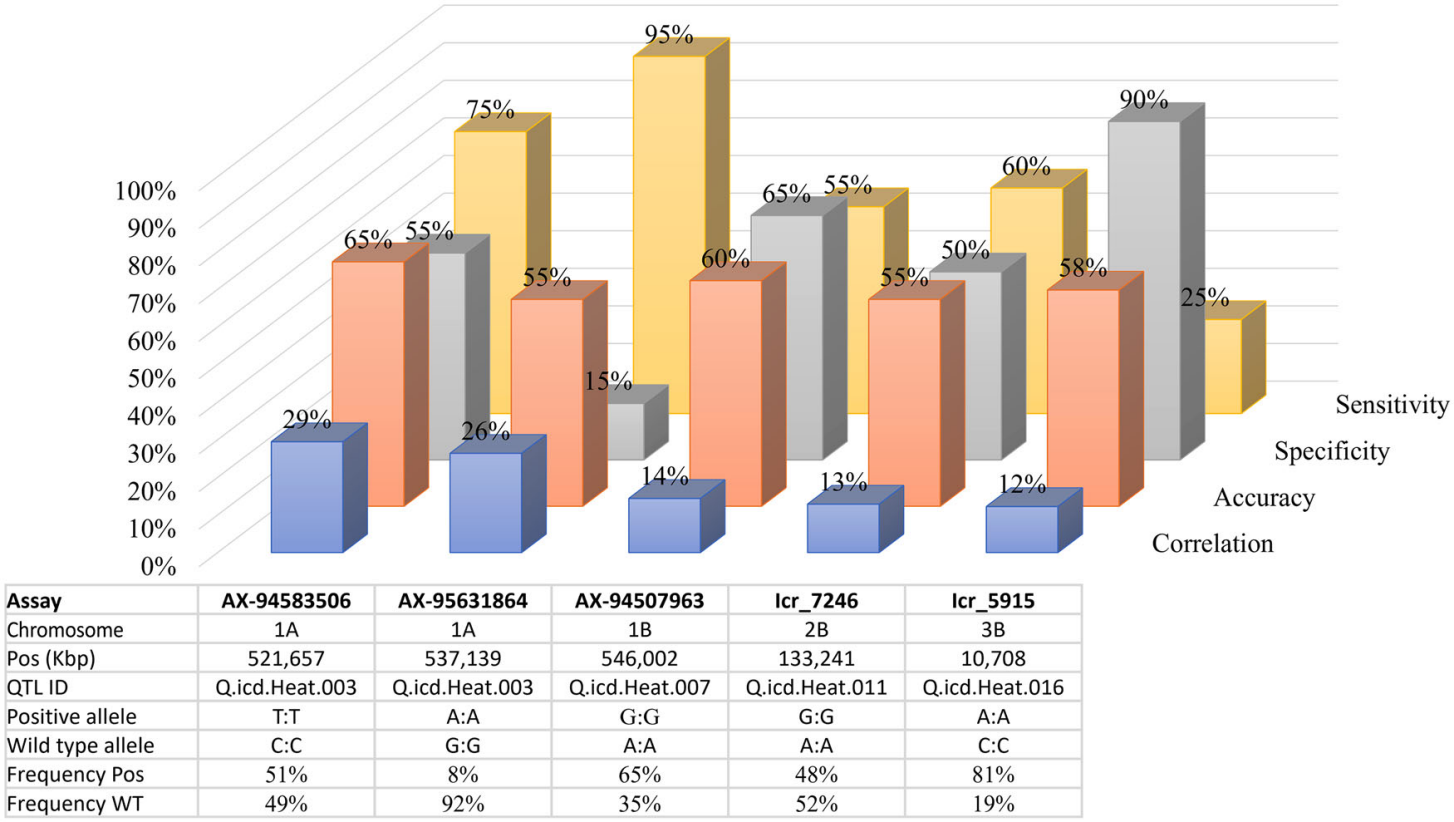
Validation of kompetitive allele specific PCR (KASP) markers on an independent set of 94 elite lines of International Center for Agricultural Research in the Dry Areas (ICARDA) tested under severe heat for grain yield. Correlation was measured between the best linear unbiased estimator (BLUE) for grain yield recorded along the Senegal River and the allelic score. Accuracy, sensitivity, and specificity were determined using only the 20 top‐performing and 20 least‐performing lines. QTL, quantitative trait locus. Pos, positive; WT, wild type.

Marker validation is a critical step required to convert QTL discovery in actual usable solutions for breeders via MAS or genomic selection (Bassi et al., 2023). In the present study, a total of 46 DNA sequences containing markers associated with the five confirmed QTLs were submitted for designing KASP primers and 36 were used for screening the validation panel. Among these, 14 resulted as polymorphic, and five markers achieved significant (*p* < 0.05) correlation to GY under heat stress (Figure 4). A marker could be validated for each of the five consistent QTLs, except for Q.icd.Heat.033 on chromosome 7A for which all tested markers were monomorphic and Q.icd.Heat.003 on chromosome 1A for which two KASPs could be validated. AX‐95631864 associated to Q.icd.Heat.003 on chromosome 1A achieved the highest correlation (29%) explaining nearly 9% of the phenotypic variation at the highest accuracy (65%). Marker AX‐94507963 underlines Q.icd.Heat.007 on chromosome 1B at 14% correlation, corresponding to 2% of the phenotypic variation at 60% accuracy and the highest overall specificity (65%). Lcr_7246 tags Q.icd.Heat.011 on chromosome 2B with 13% correlation, at 55% accuracy and 60% specificity. Lcr_5915 tags Q.icd.Heat.016 on chromosome 3B with 12% correlation, at 58% accuracy and the overall highest specificity (90%). Interestingly, the only line with positive allele at all five validated KASP was IDON43‐91 that was recorded as the overall top yielder, and the three lines with only wild type alleles at all loci were recorded among the worst 20 yielder.

## 4 DISCUSSION

### 4.1 Performances of the durum wheat panel across a north–south heat gradient

The Senegal River Basin is strongly affected by heat stress with temperatures constantly above 30°C throughout the wheat growing cycle (Sall, Kabbaj, et al., 2018). The 14 latitude parallels difference between the Moroccan northern sites and the southern ones along the River generated a 10°C temperature difference, ideal to assess the performance of the genotypes under severe temperatures. Highly significant differences could be identified for the genetic factor for GY, TKW, and Gr·spk across sites, confirming the suitability of the experiment conducted.

The heat stress environments witnessed a reduction by 46%–63% of the average GY compared to the non‐stressed conditions. Similar results were reported previously by El Hassouni et al. (2019) that also indicated a mean yield loss of 54% when the temperatures were raised by 10°C. This represents a rate of 4.6%–6.3% reduction for each 1°C temperature rise, which fall well within the 4.1%–6.4% interval suggested by Liu et al. (2016). The number of Gr·spk was the most affected trait in the heat‐stressed environments. This is in accordance with what reported by Sall, Kabbaj et al. (2018), Sall, Bassi et al. (2018), and El Hassouni et al. (2019), indicating that seed setting is the most sensitive parameter to heat stress, with a significant influence on yield. The most logical explanation for this is that the high temperatures cause a rapid dehydration of the pollen, or the accelerated dehiscence of the ovaries, or prevent the normal progression of meiosis (Draeger et al., 2020). Regardless, tolerant genotypes tend to maintain higher grain number and hence appear more capable of coping with the stress during sexual reproduction.

A positive result was the excellent yield achieved along the Senegal River reaching up to 5700 kg ha^−1^ in FAN in a cropping season that lasted only 92 days from sowing to harvest. This suggests that cultivation of wheat along the Senegal River might indeed be suitable and therefore it should be promoted to local farmers. In fact, the irrigated conditions of the Senegal River promoted the production of large kernels, which represents a critical factor for the determination of GY in durum wheat. This is in line with the study by Kumar et al. (2013) that identified TKW as a substantial contribution to GY under heat stress tolerance. However, some care should be placed when distinguishing the contribution of a trait to heat tolerance vis‐a‐vis its contribution to GY per se. In fact, our regression study identified a mild TKW contribution to GY under both heat‐stressed and non‐stressed, so it seems that this trait contributed to GY overall, but it is not specific for heat tolerance.

To better understand if traits contribute to GY overall or to heat tolerance specifically, two environmental clusters were defined based on temperature and then used to estimate HSI corrected for genotype × environment effects. Most of the investigations to date have reported HSI using simulated heat approaches—such as the contrasting of early‐ and late‐sown trials (Ayeneh et al., 2002; Kirigwi et al., 2007; Mason et al., 2011, 2010; Mohammadi et al., 2008; Pinto et al., 2010), but in this case, we contrasted actual heat‐stressed environments. HSI utilizes rate of variations in GY in stressed versus non‐stressed conditions to define a proxy for heat tolerance, as suggested by Mason et al. (2010). As such, it cannot distinguish between genotypes that maintain similar performances because of low yields under both conditions from those achieving top yields in both. For instance, the Australian cultivar Wollaroi was identified as the best for HSI, but it only achieved average yields of 3019 kg ha^−1^ under stressed conditions, nearly 800 kg less than the top yielder Berghouata1. Hence, imposing a second level of selection for the average GY across stressed environment is a better approach to find germplasm truly useful for breeders. It is then unsurprising that Berghouata1 was released in Sudan in 2020, another extremely heat‐affected environment (Tadesse et al., 2019), as a heat‐tolerant cultivar under the name Basatna.

### 4.2 Critical loci for durum wheat tolerance against severe heat

GWAS is the most popular approach for dissecting the genetic basis of complex traits (Sukumaran et al., 2015), but this approach is prone to the detection of false positives due to confounding population structure, the strong effect of phenology genes, and the tendency to overestimate significance (Yu et al., 2006; Zhang et al., 2010). Our study used a multilayer approach with three germplasm set to maximize the control of false positives. In exchange, we have certainly increased the risk of incurring a false negative. Because of the need of breeders to move ahead on solid grounds, we consider that ensuring the identification of “truly useful” alleles is more critical than losing “potentially useful” ones.

In this study, 36 QTLs were identified across all durum wheat chromosomes, all independent from the control of phenology. The use of two additional GWAS run on a subset of germplasm and an independent investigation panel confirmed five QTLs as cross‐validated. These were detected on the long arm of chromosomes 1A (Q.icd.Heat.003) and 7A (Q.icd.Heat.033), on the short arm of 2B (Q.icd.Heat.011) and 3B (Q.icd.Heat.016), and on the pericentromeric region of 1B (Q.icd.Heat.007). Important loci for heat tolerance were also identified in similar regions by Cossani and Reynolds (2012). Sukumaran et al. (2018) also identified loci associated with heat tolerance indices for GY in durum wheat overlapping with the physical position of those we identified on chromosomes 2B and 3B. Similarly, Tadesse et al. (2019) also confirmed the presence of a major QTL on chromosome 3B associated with GY in spring bread wheat tested under heat stress in Sudan and Egypt spanning the same genomic interval as Q.icd.Heat.016. Acuña‐Galindo et al. (2015) in a comprehensive meta‐analysis also identified loci involved in heat and drought tolerance in wheat on chromosomes 1B and 7A, which shows physical overlap with Q.icd.Heat.007 and Q.icd.Heat.033, respectively. Only the QTL we have identified on the long arm of chromosome 1A did not appear in our literature search and it might therefore be considered as novel to the best of our knowledge.

### 4.3 Breeding value of the identified loci in the fight against climate change

Breeding for heat tolerance remains a strategic solution to adapt crops to unpredictable environmental stresses. In that sense, the results of this study provide a good steppingstone to breed for better adaptation of durum wheat to heat stress. The analysis of additive effect of three QTLs (Q.icd.Heat.003, Q.icd.Heat.007, Q.icd.Heat.016) confirmed that the combination of positive alleles (Hap1) accounted for a 16% gain in GY under heat stress.

Among the top three entries identified for HSI and GY combined, none carried Hap1, with just Arislahn5 harboring two positive alleles (Hap3). This suggests that our conservative approach to QTL discovery, investigation, and validation has potentially prevented us from identifying additional loci responsible for Bergouata1 superiority under stress. In particular, Q.icd.Heat.033 on chromosome 7A and Q.icd.Heat.011 on chromosome 2B are good candidates to explain these top performances. Nevertheless, Icavicre having Hap1 is among the top eight entries. Also, the ICARDA's elite Ouassara harbors Hap1 and it has been released in 2020 in Senegal under the cultivar name Dioufissa. This further confirms the usefulness of Hap1 for heat tolerance. Hence, combining the alleles of Bergouata1 with those of Hap1 represents a possibility to further improve genetic gain for heat tolerance. These entries are readily available to many breeders worldwide as part of the Global Durum Panel (GDP) (Mazzucotelli et al., 2020; Oussara = GDPv2‐163 = GDPv1‐162, and Berghouata = GDPv2‐168 = GDPv1‐167) and their deployment for improving heat tolerance is highly advisable. In addition, five KASP markers (AX‐95631864, AX‐94583506, AX‐94507963, Icr_7246, and Icr_5915) have been validated in yet another independent germplasm panel of ICARDA's elites. These markers can now be used by breeders directly from LGC to follow the introgression of these alleles and ensure their rapid integration into their program.

In conclusion, this work has established a new standard for the identification of truly useful alleles when tackling by GWAS complex traits such as heat tolerance, via the deployment of three separate germplasm sets: discovery, investigation, and validation. In addition, it presents a new methodology for assessing heat tolerance using a north–south gradient that can now be deployed by others, confirming that each 1°C temperature rise reduces GY in durum wheat by 4.6%–6.3%. Furthermore, it has further highlighted the critical importance of Gr·spk to guide heat tolerance mechanism. For breeders, it delivers novel germplasm sources to favor the introgression of heat tolerance alleles, while also providing the markers to facilitate their deployment. Overall, the present work has provided a new stepping stone to facilitate the fight of the durum wheat community against climate change.

## AUTHOR CONTRIBUTIONS

**Amadou Tidiane Sall**: Conceptualization; data curation; formal analysis; investigation; resources; validation; visualization; writing—original draft; writing—review and editing. **Hafssa Kabbaj**: Data curation; formal analysis; investigation; validation; writing—original draft; writing—review and editing. **Sidi ould Ely Menoum**: Investigation; project administration; resources; writing—review and editing. **Madiama Cisse**: Conceptualization; investigation; project administration; supervision; writing—review and editing. **Mulatu Geleta**: Conceptualization; investigation; methodology; supervision; validation; writing—review and editing. **Rodomiro Ortiz**: Conceptualization; funding acquisition; investigation; methodology; project administration; resources; supervision; validation; writing—review and editing. **Filippo M. Bassi**: Conceptualization; funding acquisition; investigation; supervision; writing—original draft; writing—review and editing.

## CONFLICT OF INTEREST STATEMENT

The authors declare no conflicts of interest.

## Supporting information


**Table S1**: Complete dataset of genotypic and phenotypic information for the “Discovery panel.”
**Table S2**: Complete dataset of genotypic and phenotypic information for the “Investigation panel.”
**Table S3**: Complete dataset of genotypic and phenotypic information for the “Validation panel.”

**Table S4**. Regression of climatic factors against grain yield (GY) tested across locations by major crop growing stage.
**Table S5**. ANOVA for all traits at all environments showing the ratio of the total variance explained by each source of variation (SOV).
**Table S6**. Quantitative trait loci identified in the study by the “discovery panel” (Disc.), the “investigation panel” (Inv.) and the work with plastic tunnels (Plastic) of El Hassouni et al. (2019).

**Figure S1**: Hierarchical clustering (left) of environments by principal component analysis (right) of climatic factors significantly affecting grain yield at each individual location. TES, Tessaout; MKZ, Melk Zhar ; FAN, Fanaye ; KED, Kaedi ; 15, season 2014–2015; 16, season 2015–2016; 19, season 2018–2019; 20, season 2019–2020.

**Figure S2**: Variation for grain yield and grains per spike at each location presented as box whiskers (FAN = Fanaye, KED = Kaedi, MZ = Melk Zhar and TES = Tassaout) during the crop seasons 2014–2015 (15), 2015–2016 (16), 2018–2019 (19), and 2019–2020 (20).

## Data Availability

All generated data are made available as Table S1.
