# Supplementary material for: Durum wheat heat tolerance loci defined via a north–south gradient
Source: Plant Genome. 2023 Dec 7;17(1):e20414. doi: 10.1002/tpg2.20414 (PMC12807077; doi:10.1002/tpg2.20414)
Supplement: Supplementary file 1 [file TPG2-17-e20414-s004.docx]

Durum Wheat Heat Tolerance Loci Defined via a North-South Gradient

**Amadou Tidiane Sall , Hafssa Kabbaj, Sidi ould Ely Menoum, Madiama Cisse, Mulatu Geleta, Rodomiro Ortiz, Filippo M. Bassi**

**Table S4.** Regression of climatic factors against grain yield (GY) tested across locations by major crop growing stage

| **Crop stage** | **Climatic factor** | **Regression GY (r^2^)** |
| --- | --- | --- |
| Vegetative | Min temperature | 0.50 |
|  | Max temperature | 0.71** |
| Tillering | Min temperature | 0.53 |
|  | Max temperature | 0.62** |
| Flowering | Min temperature | 0.55 |
|  | Max temperature | 0.64** |
| Grain filling | Min temperature | 0.44 |
|  | Max temperature | 0.65** |

**, p<0.01

**Table S5.** ANOVA for all traits at all environments showing the ratio of the total variance explained by each source of variation (SOV)

| **Environment** | **SOV** | **df** | **Grain yield** | | **1000-kernels weight** | | **Grains per spike** | |
| --- | --- | --- | --- | --- | --- | --- | --- | --- |
| Fanaye 2015 | Genotype | 223 | 0.96 | ^***^ | 1.00 | ^***^ | na | - |
| Fanaye 2016 | Genotype | 223 | 0.94 | ^*^ | 0.99 | ^***^ | 0.99 | ^***^ |
| Kaedi 2015 | Genotype | 223 | 0.99 | ^***^ | 0.99 | ^***^ | na | - |
| Kaedi 2016 | Genotype | 223 | 0.95 | ^***^ | 0.94 | ^*^ | 0.97 | ^***^ |
| Combined for Senegal River | Genotype | 223 | 0.11 | ^***^ | 0.23 | ^***^ | na | - |
|  | Environments | 3 | 0.61 | ^***^ | 0.22 | ^***^ | na | - |
|  | GxE | 895 | 0.22 | ^***^ | 0.46 | ^***^ | na | - |
| Melk zher 2015 | Genotype | 223 | 0.95 | ^***^ | 0.98 | ^***^ | na | - |
| Melk zher 2016 | Genotype | 223 | 0.95 | ^**^ | 0.97 | ^***^ | 0.99 | ^***^ |
| Tessaout 2016 | Genotype | 223 | 0.94 | ^**^ | 0.99 | ^***^ | 1 | ^***^ |
| Combined for Morocco | Genotype | 223 | 0.15 | ^***^ | 0.27 | ^***^ | na | - |
|  | Environments | 2 | 0.52 | ^***^ | 0.16 | ^***^ | na | - |
|  | GxE | 662 | 0.27 | ^***^ | 0.52 | ^***^ | na | - |
| Combined for discovery panel | Genotype | 283 | 0.05 | ^***^ | 0.09 | ^***^ | 0.23 | ^**^ |
|  | Environments | 6 | 0.71 | ^***^ | 0.43 | ^***^ | 0.10 | ^**^ |
|  | GxE | 1355 | 0.20 | ^***^ | 0.45 | ^***^ | 0.55 | ^*^ |
| Fanaye 2019 | Genotype | 267 | 0.99 | ^***^ | 0.99 | ^***^ | 0.97 | ^*^ |
| Fanaye 2020 | Genotype | 123 | 0.97 | ^**^ | 0.99 | ^***^ | 0.99 | ^***^ |

***, p<0.001 ; **, p<0.01 ; *, p<0.05 ; na, not available; -, not significant

**Table S6.** Quantitative trait loci identified in the study by the “discovery panel” (Disc.), the “investigation panel” (Inv.) and the work with plastic tunnels (Plastic) of El Hassouni et al. (2019). The traits associated are reported (GY: grain yield , TKW: 1,000 kernel weight, Gr.spk: Number of grain per spike, HSI : heat susceptiblity index)band the maximum LOD recorded.

| **QTL ID** | **Trait** | **Key locus** | **Chr** | **Pos (Mbp)** | **Max-LOD**  **GY** | | | **Max-LOD TKW/Gr.spk** | | | **Max- LOD HSI** | |
| --- | --- | --- | --- | --- | --- | --- | --- | --- | --- | --- | --- | --- |
|  |  |  |  |  | **Disc.** | **Inv.** | **Plastic** | **Disc.** | **Inv.** | **Plastic** | **Disc.** | **Plastic** |
| **Q.icd.Heat.001** | TKW, Gr.spk, HSI | AX-298701935 | 1A | 4,688 | - | - | - | 6.9 | - | - | 7.1 | - |
| **Q.icd.Heat.002** | GY, TKW, Gr.spk, HSI | AX-95015657 | 1A | 206,032 | 3.2 | - | 3.4 | 3.9 | - | 3.4 | 7.0 | 3.4 |
| **Q.icd.Heat.003** | GY, TKW, Gr.spk, HSI | AX-94693391 | 1A | 390,755 | 2.7 | 3.7 | 3.4 | 7.5 | 3.6 | 3.4 | 7.0 | 3.4 |
| **Q.icd.Heat.004** | TKW, Gr.spk, HSI | AX-94498055 | 1B | 314 | - | - | - | 7.1 | - | - | 8.5 | - |
| **Q.icd.Heat.005** | GY,TKW, Gr.spk, HSI | AX-95186349 | 1B | 168,237 | 2.9 | - | - | 8.4 | 3.6 | - | 8.9 | - |
| **Q.icd.Heat.006** | GY, TKW, Gr.spk, HSI | AX-95016115 | 1B | 394,495 | 3.2 | - | - | 7.8 | - | - | 5.8 | - |
| **Q.icd.Heat.007** | GY, TKW, Gr.spk, HSI | AX-94620168 | 1B | 541,901 | 2.8 | 3.5 | - | 3.9 | - | 3.4 | 4.6 | - |
| **Q.icd.Heat.008** | GY, TKW, Gr.spk, HSI | AX-94464525 | 2A | 41,763 | 3.1 | - | - | 3.7 | - | - | 6.5 | - |
| **Q.icd.Heat.009** | TKW, Gr.spk, HSI | AX-94529895 | 2A | 408,246 | - | - | - | 3.2 | - | - | 3.3 | - |
| **Q.icd.Heat.010** | GY, TKW, Gr.spk, HSI | AX-94459397 | 2A | 617,694 | 3.2 | - | 3.1 | 5.3 | - | 3.1 | 6.4 | 3.1 |
| **Q.icd.Heat.011** | GY, TKW, Gr.spk, HSI | AX-94598102 | 2B | 406 | - | 3.3 | 3.7 | 7.5 | - | 3.7 | 8.6 | - |
| **Q.icd.Heat.012** | GY, TKW, Gr.spk, HSI | AX-95070074 | 2B | 386,346 | - | - | 3.7 | 7.7 | - | 3.7 | 6.5 | - |
| **Q.icd.Heat.013** | GY, TKW, Gr.spk, HSI | AX-94823565 | 2B | 576,573 | - | - | 3.7 | 7.7 | - | 3.7 | 8.9 | - |
| **Q.icd.Heat.014** | TKW, Gr.spk, HSI | AX-94510991 | 3A | 1,805 | - | - | - | 8.7 | - | 3.4 | 8.6 | - |
| **Q.icd.Heat.015** | GY, TKW, Gr.spk, HSI | AX-95632723 | 3A | 562,421 | 3.4 | - | - | 6.9 | - | 3.4 | 9.4 | - |
| **Q.icd.Heat.016** | GY, TKW, Gr.spk, HSI | AX-94877000 | 3B | 2,652 | 3.0 | - | - | 3.9 | 2.9 | 3.4 | 7.1 | - |
| **Q.icd.Heat.017** | TKW, Gr.spk, HSI | AX-94474764 | 3B | 419,880 | - | - | - | 6.4 | - | - | 8.5 | - |
| **Q.icd.Heat.018** | GY, TKW, Gr.spk, HSI | AX-95148741 | 3B | 512,173 | 3.2 | - | - | 7.1 | - | 3.4 | 8.4 | - |
| **Q.icd.Heat.019** | GY, TKW, Gr.spk, HSI | AX-94524164 | 4A | 70,275 | 3.5 | - | - | 3.9 | - | - | 5.8 | - |
| **Q.icd.Heat.020** | GY, TKW, Gr.spk, HSI | AX-94649014 | 4A | 537,345 | 4.0 | - | - | 7.3 | 3.1 | - | 5.5 | - |
| **Q.icd.Heat.021** | GY, TKW, Gr.spk, HSI | AX-94440259 | 4B | 9,122 | 2.8 | 2.9 | - | 3.3 | - | - | 6.5 | - |
| **Q.icd.Heat.022** | GY, TKW, Gr.spk, HSI | AX-94496513 | 4B | 484,213 | 3.2 | - | - | 6.9 | - | - | 9.0 | - |
| **Q.icd.Heat.023** | GY, TKW, Gr.spk, HSI | AX-94920631 | 5A | 10,352 | 4.5 | - | - | 9.7 | - | 3.4 | 7.1 | - |
| **Q.icd.Heat.024** | GY, TKW, Gr.spk, HSI | AX-94455614 | 5A | 418,786 | - | - | 4.9 | 7.7 | - | 4.2 | 7.4 | 4.9 |
| **Q.icd.Heat.025** | GY, TKW, Gr.spk, HSI | AX-94490702 | 5B | 24 | 4.1 | - | - | 9.7 | 2.8 | - | 7.1 | - |
| **Q.icd.Heat.026** | GY, TKW, Gr.spk, HSI | AX-95258852 | 5B | 497,193 | - | - | 4.2 | 3.9 | - | 4.2 | 7.3 | 4.2 |
| **Q.icd.Heat.027** | GY, TKW, Gr.spk, HSI | AX-95630503 | 6A | 1,885 | 2.9 | 3.0 | - | 6.9 | - | - | 6.5 | - |
| **Q.icd.Heat.028** | GY, TKW, Gr.spk, HSI | AX-95089938 | 6A | 498,783 | 2.8 | - | - | 8.2 | - | - | 4.8 | - |
| **Q.icd.Heat.029** | GY, TKW, Gr.spk, HSI | AX-94406711 | 6B | 28,677 | 3.0 | - | 3.2 | 7.7 | - | 3.4 | 5.7 | 3.7 |
| **Q.icd.Heat.030** | GY, TKW, Gr.spk, HSI | AX-94563631 | 6B | 542,513 | 2.7 | - | - | 7.7 | - | - | 5.7 | - |
| **Q.icd.Heat.031** | TKW, Gr.spk, HSI | AX-94667805 | 7A | 8,306 | - | - | - | 6.9 | - | 3.4 | 5.3 | - |
| **Q.icd.Heat.032** | GY, TKW, Gr.spk, HSI | AX-94759589 | 7A | 450,732 | 3.3 | - | - | 7.7 | 3.4 | - | 8.3 | - |
| **Q.icd.Heat.033** | GY, TKW, Gr.spk, HSI | AX-94986625 | 7A | 660,348 | 3.0 | - | - | 4.1 | 3.4 | 3.6 | 4.3 | - |
| **Q.icd.Heat.034** | TKW, Gr.spk, HSI | AX-94498463 | 7B | 53,923 | - | - | - | 3.7 | - | - | 7.3 | - |
| **Q.icd.Heat.035** | GY, TKW, Gr.spk, HSI | AX-95257383 | 7B | 312,626 | 3.0 | - | - | 2.8 | - | - | 5.2 | - |
| **Q.icd.Heat.036** | GY,TKW, Gr.spk, HSI | AX-94964989 | 7B | 514,034 | 3.0 | - | - | 4.2 | - | - | 8.3 | - |
